# Prevalence of human leukocyte antigen HLA-B*57:01 in individuals with HIV in West and Central Africa

**DOI:** 10.1186/s12865-021-00427-7

**Published:** 2021-07-22

**Authors:** Malewe Kolou, Armel Poda, Zelica Diallo, Esther Konou, Tatiana Dokpomiwa, Jacques Zoungrana, Mounerou Salou, Lionèle Mba-Tchounga, André Bigot, Abdoul-Salam Ouedraogo, Marielle Bouyout-Akoutet, Didier K. Ekouevi, Serge P. Eholie

**Affiliations:** 1 Laboratoire de Biologie Moléculaire et d’Immunologie (BIOLIM), Université de Lomé, Faculté des Sciences de la santé, Lomé, Togo; 2 Department of Infectious Diseases, Université Nazi Boni, Bobo-Dioulasso, Burkina Faso; 3 Département de Dermatologie et Maladies Infectieuses, Université Félix Houphouët-Boigny, UFR des Sciences Médicales, Abidjan, Côte d’Ivoire; 4 Department of Pharmacy, Faculty of Health Science, University of Abomey-Calavi, Cotonou, Benin; 5 Programme PACCI, Site de recherche ANRS de Côte d’Ivoire, Abidjan, Côte d’Ivoire; 6 Department of Medical Bacteriology and Virology, Université Nazi BONI, CHU Souro Sanou, Bobo-Dioulasso, Burkina Faso; 7 Department of Parasitology, Mycology and Tropical Medicine, Université des Sciences de la Santé, Libreville, Gabon; 8 Département de santé Publique, Université de Lomé, Faculté des Sciences de la santé, Lomé, Togo; 9 Centre Inserm 1219 & Institut de Santé Publique d’épidémiologie et de développement, Université de Bordeaux, Bordeaux, France

**Keywords:** Abacavir, Antiretroviral, HLA-B*57:01, HIV infection, Prevalence, West, And Central Africa

## Abstract

**Background:**

The presence of the human leukocyte antigen HLA-B*57:01 is associated with the development of a hypersensitivity reaction to abacavir (ABC). Limited data exist on HLA-B*57:01 prevalence in individuals with HIV-1 in Africa. This study aimed to estimate HLA-B*57:01 prevalence in individuals with HIV-1 in West and Central Africa.

**Methods:**

A cross-sectional study was conducted in four countries in West and central Africa (Burkina-Faso, Côte d’Ivoire, Gabon, and Togo) from January 2016 to February 2020 to determine the status of HLA-B*57:01 in adults with HIV-1. The presence of HLA-B*57:01 was determined by using Single Specific Primer-Polymerase Chain Reaction (SSP-PCR) in blood samples. Prevalence rates were stratified based on country.

**Results:**

A total of 4016 (69.8% women) individuals with HIV were enrolled. Their median age was 45, and the interquartile range was 38–52. We included 500 (12.4%) patients in Burkina-Faso, 1453 (36.2%) in Côte d’Ivoire, 951 (23.7%) in Gabon, and 1112 (27.7%) in Togo. The overall HLA-B*57:01 prevalence was 0.1% [95% CI: 0.0–0.2%]. The prevalence of HLA-B*57:01 was similar according to the four countries. Only one case was reported in each country except Togo, with no cases.

**Conclusions:**

HLA-B*57:01 prevalence is low in individuals with HIV in West and central Africa, and there is no difference among countries. This study does not confirm the utility of HLA-B*57:01 allele testing for abacavir use in this region.

## Background

According to UNAIDS, new HIV infections have been reduced by 23% since 2010, largely due to a substantial 38% decrease in Eastern and Southern Africa [1]. Despite this reduction in new HIV infections, there were 690,000 AIDS-related deaths in 2019 [1]. The rollout of combination antiretroviral therapy (ART) has significantly changed the natural history of HIV infection [2]. Advances in treatment strategies have led to a spectacular drop in the mortality rate of people living with HIV (PLHIV) worldwide [3]. However, of the 38 million people living with HIV worldwide, only 25.4 million are on treatment [1]. Abacavir, a nucleoside HIV reverse transcriptase inhibitor, is one of the drugs used in the management of HIV infection. It is a key component of the first-line antiretroviral (ART) regimens recommended by the US Department of Health and Human Services (DHHS) [4]. A randomized, double-blind study from 2015 called for positioning the dolutegravir/abacavir/lamivudine regimen as a first-line option for treatment-naïve HIV-positive patients [5]. Indeed, of the 833 randomized participants, 71% in the dolutegravir/abacavir/lamivudine group and 63% in the efavirenz/tenofovir/emtricitabine group maintained viral loads of < 50 copies per milliliter up to 144 weeks after initiation of treatment (*P* = 0.01) [5]. The higher efficacy was mainly due to fewer dropouts due to adverse events in the dolutegravir/abacavir/lamivudine group [dolutegravir/abacavir/lamivudine group, 13 (3%); efavirenz/tenofovir/emtricitabine group, 48 (11%)] [5]. Several studies have provided clear benefits and recommended abacavir-based treatment regimens [6–8]. According to the recent World Health Organization (WHO) guidelines in low-income countries, especially in West and Central Africa, abacavir is recommended as an alternative to first-line regimens in children and, in special circumstances in adults [9].

The main limitation to the use of abacavir is a frequent, early and potentially fatal Type IV hypersensitivity reaction [10]. Data from several clinical trials suggest that an abacavir-induced hypersensitivity reaction occurs in 2.3–9% of patients exposed to abacavir [11–13]. In a cohort of 14,310 individuals with HIV-1 examined in 93 centers across Europe, Israel and Argentina, 2101 (64.1%) were forced to abandon abacavir treatment [14]. For 167 (5.1%) patients, the reason for discontinuing treatment was the occurrence of a hypersensitivity reaction, and the overall incidence of hypersensitivity reactions in the study was estimated at 22.1 (95% confidence interval [CI] 18.7–25.4) per 100 person-years [14].

Human leukocyte antigen (HLA)-B*57:01, an allele of the major histocompatibility complex, has been strongly associated with the risk of an abacavir hypersensitivity reaction [15]. A prospective, randomized, multicenter, double blind, prospective study has shown that screening for HLA-B*57:01 can reduce the incidence of hypersensitivity reactions to abacavir [16].. Several studies have been conducted to estimate the prevalence of HLA-B*57:01 in many populations around the world. A meta-analysis by the Clinical Pharmacogenetics Implementation Consortium (CPIC) of data published between 1950 and 2011 allowed the mapping of the distribution of HLA-B*57:01 in different ethnic populations [17]. The combined analyses covered 35,630 Europeans, 1321 South Americans, 8570 Africans, 1029 Middle Easterners, 3391 Mexicans and 12,501 Asians. Europeans recorded the highest frequency of HLA-B*57:01, with proportions up to 14.1% [17]. Among South Americans and Mexicans, 2.6 and 2.2%, respectively, were carriers of HLA-B*57:01 [17]. This proportion was 2.5% for Middle East residents and 12.6% for Asian residents [17]. African populations recorded the lowest prevalence with an estimated overall frequency of 1.0% [17].

The majority of HLA-B*57:01 prevalence surveys among people from Africa are conducted among African Americans [18–21]. However, some studies have been carried out in sub-Saharan Africa, notably in Nigeria, Kenya, South Africa and Uganda [22–26], but data on the distribution of HLA-B*57:01 in the population of Sub-Saharan Africa remain scarce.

In Burkina Faso, according to a study carried out in 2014 among 51 HIV sero-discordant couples, 78% of individuals with HIV were carriers of the HLA-B*57:01 gene [27]. This high frequency, in contrast to what is generally reported in African populations, has led the authors of this Burkinabe study to recommend large cohort studies to confirm their results [27]. In the Ivory Coast, Gabon and Togo, to our knowledge, there are no data on the prevalence of the HLA-B*57:01 gene. The objective of this study was to estimate the prevalence of the HLA-B*57:01 gene in individuals with HIV in four West and Central African countries (Burkina Faso, Ivory Coast, Gabon and Togo) and to measure the association between HLA-B*57:01 gene carrying and ethnic subgroups in West and Central Africa.

## Methods

**Fig. 1:**
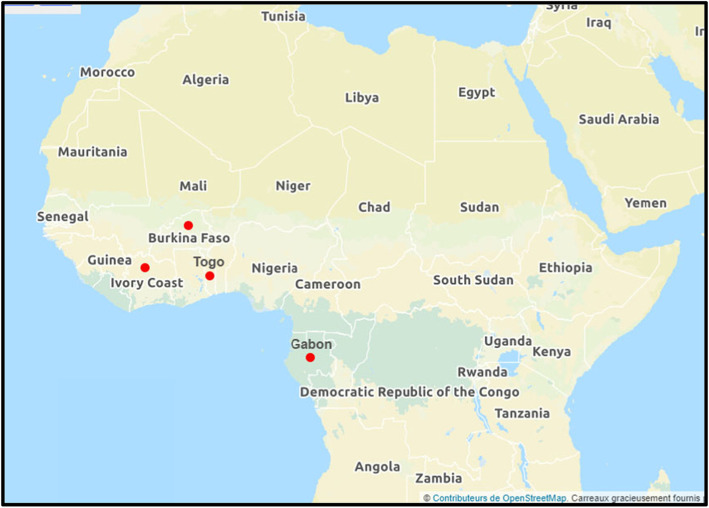
Participating countries located in West and Central Africa. Figure 1 shows location of participating countries across West and Central Africa. These countries appear with red mark on the map. The figure was created by us, using Openstreetmap and Adobe Photoshop CC 2019 software version 20.0.4 by Adobe Systems Incorporated**©** (https://www.adobe.com/fr/products/photoshop.html#)

A multicounty, multicentric cross-sectional study was conducted from January 2016 to February 2020 in four countries in Central and West Africa: Togo, Burkina Faso, Ivory Coast and Gabon (Fig. 1).

### Study population

Participants were recruited from clinical centers chosen based on existing partnerships and centers’ involvement in clinical research projects on HIV. Those clinical centers were in four countries. In Burkina Faso, the clinical center was the Day Hospital, University Hospital Sourou Sanon (Bobo-Dioulasso); The Support, Research and Training Center (CePReF) (Abidjan); the Infectious and Tropical Diseases Service (SMIT); Treichville University Hospital (Abidjan); and the medical center for monitoring blood donors (CMSDS), CNTS, Treichville (Abidjan), which was included in Côte d’Ivoire. Participants from Gabon were enrolled at the Mélène Hospital in Libreville, the Parasitology Mycology Laboratory at the Faculty of Medicine of the University of Health Sciences and at the outpatient Treatment Center (CTA) of Oyem and Koulamoutou. Finally, in Togo, two centers were involved: the day hospital of the infectious diseases department of the Sylvanus Olympio University Hospital and the Laboratory of Molecular Biology and Immunology of the Faculty of Health Sciences of the University of Lomé (BIOLIM-FSS/UL).

Participants were eligible to participate in the study if the following four criteria were met: (i) infected with HIV, regardless of subtype (HIV-1, HIV-2, HIV-1 + 2); (ii) being 18 years of age or older; (iii) being from West or Central Africa; (iv) having consulted in one of the study centers and residing in the study city; and (v) having given free, informed and written consent, signed by the participant and the investigating physician on the day of inclusion and before any sampling required by the study.

### Sample size estimation

The sample size of participants was calculated using a single proportion population formula with a 95% confidence level. We hypothesized that the prevalence of HLA-B*57:01 allele among black Africans is less than 1% with a 1% margin error. Considering a 10% nonresponse rate, the minimum number of participants per country was estimated at 418 and a minimum sample size was estimated at 1672 for the four countries participating to the study.

### Data collection

After eligibility screening and written informed consent, sociodemographic characteristics and HIV epidemiological data were collected using a standardized questionnaire. The questionnaire was administered by a trained study team during a face-to-face interview. After the interview, information on biological variables and ART treatment was obtained from another data source (biological database and pharmacy). Sociodemographic and clinical characteristics (age, sex, nationality, ethnic group, WHO clinical stage, history of antiretroviral therapy) were collected. Biological variables collected were absolute CD4 count, HIV type, HLA type and presence of HLA-B*57:01. From each patient, whole blood samples were collected at the elbow fold in two EDTA tubes and were used for the detection of the HLA-B*57:01 allele.

### Laboratory procedures

#### Sample preparation and nucleic acid extraction

Blood samples were centrifuged at 3500 tours/min. Buffy coats were collected, aliquoted and stored at − 80 °C. DNA was extracted from buffy coat samples using the QIAamp® DNA Mini kit according to the manufacturer’s instructions. The DNA eluate was stored at − 80 °C and sent to *Laboratoire d’Histocompatibilité des Cliniques Universitaire Saint Luc* in Belgium, where HLA-B*57:01 typing was performed.

#### HLA-B*57:01 typing

A qPCR-SSP DNA generic typing test (LinkSēq™ HLA-B*57:01-ONE LAMBDA) was used. Specifically, generic real-time PCR was performed in 96-well plates. The test separately detected HLA-B*57:01 and closely related HLA-B57 alleles (HLA-B*57:02/HLA-B*57:03) for each sample. As recommended by the manufactured protocol, DNA samples were added immediately after isolation to each well of the tray to the Taq polymerase reconstituted with a dNTP-buffer mix (Micro SSP D-mix). Each typing tray included a negative control reaction tube that detects the presence of the internal control PCR product. PCR amplification was carried out according to standard procedures (One Lambda, Inc.) in LightCycler® 480 Real-Time PCR System-Roche. SureTyper software was then used to analyze and interpret the melt curves generated from each well, and the results were thus obtained.

### Statistical analysis

Data describing clinical and demographic patient characteristics were summarized using medians with interquartile ranges (IQRs) for continuous variables and frequencies and proportions for categorical variables. Prevalence was estimated with their 95% confidence interval (95% CI). Fisher’s exact tests was used to compare categorical variables. All analyses were performed using R© version 3.4.3 software, and the level of significance was set at 5%.

### Ethical considerations

Ethical approval was obtained from the ethics committees in Burkina Faso, Ivory Coast, Gabon and Togo before the start of the inclusion. Potential participants were informed about the study purpose and procedures, potential risks and protections. Those willing to participate were invited to sign a consent prior to participation.

## Results

### Population characteristics

A total of 4016 HIV-positive patients from four countries were included in this study. Table 1 presents the number of patients enrolled in each country in West and Central Africa.

**Table 1:** Number of participant per countries

	Frequency	Proportion (%)
**Côte d’Ivoire**	1453	36.2
**Gabon**	951	23.7
**Togo**	1112	27.7
**Burkina Faso**	500	12.4
**Total**	4016	100.0

The median age of the participants was 45 years (IQR = [38–52]), and more than two-thirds (69.8%) were female. Most participants were 40–49 years old (38.0%), and 46.9% had at least a secondary school level of education. The majority of patients were on ART (*n* = 3510; 87.4%), and among them, 117 (3.3%) were on ART containing abacavir.

More than two-thirds (62.4%) had been diagnosed with HIV for more than 5 years (at the time of the survey). A higher proportion of them (90.9%) had HIV-1 status, and approximately one-quarter (26.3%) were at WHO stage I. Other demographic, clinical, and laboratory characteristics of all participants by country are presented in Table 2.

**Table 2:** Demographic, clinical and laboratory characteristics of individuals with HIV in West and Central Africa (2016–2020)

	Côte d’Ivoire	Gabon	Togo	Burkina Faso	Total
	(***n*** = 1453)	(***n*** = 951)	(***n*** = 1112)	(***n*** = 500)	(***n*** = 4016)
**Sex**					
Male	483 (33.2)	303 (31.9)	329 (29.6)	96 (19.2)	1211 (30.2)
Female	970 (66.8)	648 (68.1)	783 (70.4)	404 (80.8)	2805 (69.8)
**Age (years), Median [IQR]**	46 [40–53]	44 [36–52]	44 [37–51]	44 [38–51]	45 [38–52]
**Age (years)**					
< 20	3 (0.2)	21 (2.2)	12 (1.1)	1 (0.2)	37 (0.9)
20–29	55 (3.8)	88 (9.3)	76 (6.8)	19 (3.8)	238 (5.9)
30–39	283 (19.5)	238 (25.0)	267 (24.0)	142 (28.4)	930 (23.2)
40–49	584 (40.2)	322 (33.9)	426 (38.3)	196 (39.2)	1528 (38.0)
50–59	399 (27.5)	198 (20.8)	256 (23.0)	118 (23.6)	971 (24.2)
≥ 60	129 (8.9)	84 (8.8)	75 (6.7)	24 (4.8)	312 (7.8)
**Education level**					
None	499 (34.3)	186 (19.6)	199 (17.9)	202 (40.4)	1086 (27.0)
Primary	301 (20.7)	148 (15.6)	390 (35.1)	209 (41.8)	1048 (26.1)
Secondary	435 (29.9)	448 (47.1)	454 (40.8)	84 (16.8)	1421 (35.4)
University	218 (15.0)	169 (17.8)	69 (6.2)	5 (1.0)	461 (11.5)
**Time since HIV diagnosis (years)**					
< 1	78 (5.4)	135 (14.2)	126 (11.3)	20 (4.0)	359 (8.9)
[1–3]	132 (9.1)	170 (17.9)	160 (14.4)	43 (8.6)	505 (12.6)
[3–5]	155 (10.7)	147 (15.5)	144 (12.9)	52 (10.4)	498 (12.4)
≥ 5	1065 (73.3)	389 (40.9)	666 (59.9)	384 (76.8)	2504 (62.4)
Missing	23 (1.6)	110 (11.6)	16 (1.4)	1 (0.2)	150 (3.7)
**CD4 cell count, Median [IQR]**	230 [127–367]	237 [138–440]	169 [84–297]	227 [110–333]	210 [106–340]
**Baseline CD4 cell count (cells/mm3)**					
< 200	563 (38.7)	78 (8.2)	549 (49.4)	201 (40.2)	1391 (34.6)
[200–350]	407 (28.0)	56 (5.9)	243 (21.9)	148 (29.6)	854 (21.3)
[350–500]	177 (12.2)	25 (2.6)	111 (10.0)	58 (11.6)	371 (9.2)
≥ 500	188 (12.9)	40 (4.2)	52 (4.7)	47 (9.4)	327 (8.1)
Missing	118 (8.1)	752 (79.1)	157 (14.1)	46 (9.2)	1073 (26.7)
**HIV status**					
HIV-1	1370 (94.3)	734 (77.2)	1078 (96.9)	469 (93.8)	3651 (90.9)
HIV-2	37 (2.5)	4 (0.4)	7 (0.6)	17 (3.4)	65 (1.6)
HIV-1 + 2	34 (2.3)	54 (5.7)	1 (0.1)	14 (2.8)	103 (2.6)
Missing	12 (0.8)	159 (16.7)	26 (2.3)	0 (0.0)	197 (4.9)
**WHO stage**					
Stage I	586 (40.3)	15 (1.6)	323 (29.0)	134 (26.8)	1058 (26.3)
Stage II	194 (13.4)	24 (2.5)	298 (26.8)	106 (21.2)	622 (15.5)
Stage III	236 (16.2)	31 (3.3)	180 (16.2)	179 (35.8)	626 (15.6)
Stage IV	30 (2.1)	1 (0.1)	87 (7.8)	25 (5.0)	143 (3.6)
Missing	407 (28.0)	880 (92.5)	224 (20.1)	56 (11.2)	1567 (39.0)
**Abacavir based regimen**^a^					
No	1277 (98.6)	565 (87.9)	1053 (98.2)	498 (99.6)	3393 (96.7)
Yes	18 (1.4)	78 (12.1)	19 (1.8)	2 (0.4)	117 (3.3)

^a^Among 3510 on ARV treatment

### Prevalence of HLA-B57 and HLA- B*57:01 per country

Of the 4016 patients who underwent HLA typing, 259 were positive for HLA-B57, allowing the overall prevalence of the HLA-B57 allele to be estimated at 6.4% (95% CI: 5.7–7.3). There was no significant difference in the prevalence of the HLA-B057 allele according to country (*p* = 0.238), sex (*p* = 0.078), or age (*p* = 0.276) (Table 3).

**Table 3:** Prevalence of HLA-B57 and HLA-B*57:01 per country in West and Central Africa (2016–2020)

	N	HLA-B57^**+**^			HLA-B*57:01^**++**^		
		n	%	95 CI%	n	%	95CI%
Côte d’Ivoire	1453	107	7.4	[6.1–8.8]	1	0.1	[0.0–0.4]
Gabon	951	51	5.4	[4.0–7.0]	1	0.1	[0.0–0.6]
Togo	1112	72	6.5	[5.1–8.1]	0	0.0	[0.0–0.3]
Burkina Faso	500	29	5.8	[3.9–8.2]	1	0.2	[0.0–0.1]
Total	4016	259	6.4	[5.7–7.3]	3	0.1	[0.0–0.2]

^**+**^*p*-value = 0.238; ^**++**^*p*-value = 0.571

*95 CI%* 95% Confidence Interval

### Prevalence HLA- B*57:01 per country

Among 259 patients with the HLA-B57 allele, three were positive for the HLA-B*57:01 allele. The overall prevalence in the population was 0.1% (95% CI: 0.0–0.2). One case was reported in Côte d’Ivoire, Gabon and Burkina Faso, and no case was reported in Togo (Table 3).

## Discussion

To our knowledge, this study is the first large multicountry study conducted in four countries in the West (*n* = 3) and Central (*n* = 1) African regions of the prevalence of the HLA-B*57:01 allele among individuals with HIV. According to the latest epidemiological data, West and Central Africa is home to 4.9 million people living with HIV. HIV prevalence among adults is 1.4%, which is relatively low compared to East and Southern Africa. In this region, the number of people accessing treatment rose significantly from 860,000 in 2010 to 2.9 million in 2019. HLA-B*57:01 is a generic marker of clinical importance used in several countries, specifically in developed countries, to decrease abacavir-related hypersensitivity reactions. Guidelines in North America and Europe recommend routine screening for HLA-B*57:01 prior to initiation of abacavir therapy [28]. Ideally, since HLA-B*57:01 varies among different populations, it is ascertaining HLA-B*57:01 prevalence before decoding or not performing systematic screening before initiating abacavir-based ART regimens. In our study, we screened HIV-positive patients on ART or not for HLA-B*57:01 carriage.

We estimated an overall HLA-B*57:01 prevalence rate of 0.07%, ranging from 0% in Togo to 0.2% in Burkina-Faso. A similar survey was conducted in Nigeria, located in the West African region between April 2016 and April 2017 in five HIV treatment facilities. In this study, 1504 adults were enrolled. Of these, 132 (9.1%) were HLA-B57 positive using a nonspecific low-resolution HLA-B*57:01 primer mix. On further analysis, none of the 132 samples (0%) had the HLA-B*57:01 allele [22]. Another survey was conducted in a different population and reported a high prevalence of HLA-B*57:01. This is the case in Colombia, with a prevalence rate of 2.7% in Colombian HIV-infected individuals. The prevalence was 4% for whites, 2.6% for other races, and 1.9% for Afro-Colombians [29]. Other studies have confirmed a low prevalence of HLA-B*57:01 in the African population. In the United States, there is a lower frequency of the HLA-B*57:01 allele in African Americans, with a reported frequency between 2.3 and 4% [20, 30, 31]. Among White subjects, prevalence was 7.93%. Among black subjects, only two (both Ugandan) were HLA-B*57:01 positive, giving a rate of 0.26% [32].

Our study had a few limitations. We did not conduct an analysis based on race in our survey. Other races are indeed rare in individuals with HIV in West and central Africa, and this variable is not collected routinely in databases. Further studies should explore including race (i.e., mestizo), as in our sample, one person in Gabon with the mestizo race was screened positive. Some studies have reported that the heterogeneity of HLA-B*57:01 prevalence is mostly dependent upon race and ethnicity heritage [16, 33]. Another limitation is that we did not perform logistic regression with the low prevalence observed to identify factors associated with the presence of HLA-B*57:01. Another limitation to the study is that HLA typing were performed on patients mostly already on ART. This design could induce some bias towards excluding patients already having had hypersensitivity reaction to abacavir. Finally, HLA-B*57:01-negative patients starting abacavir-containing regimens (3%) were not followed up; therefore, the incidence of HSR was not assessed.

Since abacavir is included in the first-line regimen, the question of hypersensitivity to abacavir should be important to document. However, to our knowledge, there are no guidelines for screening patients before ART initiation in Africa. Before abacavir should be wisely used in Africa, it is necessary to explore the prevalence of HLA-B*57:01. The application of routine HLA-B*57:01 testing to other racial populations deserves further investigation. In these four countries, antiretroviral therapy is available and free of charge for patients. Currently, the first-line regimen includes 2 NRTIs and dolutegravir in accordance with the WHO guidelines or 2 NRTIs and efavirenz. An optimized NRTI backbone should be used, such as zidovudine (AZT), tenofovir or abacavir (ABC), and vice versa.

In addition, HLA-B*57:01 screening was not performed routinely because of the lack of equipment and reference laboratories specializing in immunologic tests in the four countries. This is the main reason all-immunological analyses were performed in Belgium, which is specialized in this analysis to avoid heterogenicity between the tests.

Recently, the value of HLA-B*57:01 screening prior to prescribing abacavir is a concern in areas where its prevalence is low [34, 35]. In the context of low-income countries where pharmacogenetic screening is not available and based on this result with a low prevalence of HLA-B*57:01, we could not recommend the testing of HLA-B*57:01. Despite the low prevalence of HLA-B*57:01, clinical follow-up of patients starting an abacavir-based regimen is mandatory. It is therefore important to educate the patient about the possible symptoms of a hypersensitivity reaction, especially at the beginning of treatment, so that the patient can easily identify them and return for further consultation. Any suspicion of hypersensitivity to abacavir should lead to discontinuation of treatment and the initiation of rigorous clinical follow-up [36, 37]. Screening for HLA-B*57:01 prior to initiation of any therapy with abacavir has been shown to be beneficial [38, 39].

## Conclusion

HLA-B*57:01 is rare in people living with HIV, in the four countries participating to this survey, and there are no significant differences among countries. HLA-B*57:01 screening may not be a cost-effective strategy to reduce the risk associated with abacavir hypersensitivity in these countries. Indeed, the prevalence of HLA-B*57:01 allele positivity was low in the study population. These results cannot be generalized to the West and Central African region as prevalence may be much higher in other countries and among other ethnicities in the same regions of Africa.

However, despite the low prevalence of the HLA-B*57:01 allele, rigorous monitoring should be maintained in any patient on ABC since the possibility of occurrence of HSR to this drug should not be definitively ruled out.

## Data Availability

The datasets used and/or analysed during the current study are available from the corresponding author upon reasonable request.
